# Investigating Oral Microbiome Profiles in Children with Cleft Lip and Palate for Prognosis of Alveolar Bone Grafting

**DOI:** 10.1371/journal.pone.0155683

**Published:** 2016-05-18

**Authors:** Luwei Liu, Qian Zhang, Jiuxiang Lin, Lian Ma, Zhibo Zhou, Xuesong He, Yilin Jia, Feng Chen

**Affiliations:** 1 Department of Orthodontics, Peking University School and Hospital of Stomatology, Beijing, P.R. China; 2 Central Laboratory, Peking University School and Hospital of Stomatology, Beijing, P.R. China; 3 Department of Oral and Maxillofacial Surgery, Peking University School and Hospital of Stomatology, Beijing, P.R. China; 4 School of Dentistry, University of California Los Angeles, Los Angeles, United States of America; University of Illinois at Urbana-Champaign, UNITED STATES

## Abstract

In this study, we sought to investigate the oral microbiota structure of children with cleft lip and palate (CLP) and explore the pre-operative oral bacterial composition related to the prognosis of alveolar bone grafting. In total, 28 patients (19 boys, 9 girls) with CLP who were scheduled to undergo alveolar bone grafting for the first time were recruited. According to the clinical examination of operative sites at the third month after the operation, the individuals were divided into a non-inflammation group (n = 15) and an inflammation group (n = 13). In all, 56 unstimulated saliva samples were collected before and after the operation. The v3-v4 hypervariable regions of the *16S rRNA* gene were sequenced using an Illumina MiSeq sequencing platform. Based on the beta diversity of the operational taxonomic units (OTUs) in the inflammation and non-inflammation samples, the microbial variation in the oral cavity differed significantly between the two groups before and after the operation (P < 0.05). Analysis of the relative abundances of pre-operative OTUs revealed 26 OTUs with a relative abundance higher than 0.01%, reflecting a significant difference of the relative abundance between groups (P < 0.05). According to a principal component analysis of the pre-operative samples, the inflammation-related OTUs included *Tannerella sp*., *Porphyromonas sp*., *Gemella sp*., *Moraxella sp*., *Prevotella nigrescens*, and *Prevotella intermedia*, most of which were enriched in the inflammation group and showed a significant positive correlation. A cross-validated random forest model based on the 26 different OTUs before the operation was able to fit the post-operative status of grafted sites and yielded a good classification result. The sensitivity and specificity of this classified model were 76.9% and 86.7%, respectively. These findings show that the oral microbiota profile before alveolar bone grafting may be related to the risk of post-operative inflammation at grafted sites.

## Introduction

Alveolar cleft, a symptom often occurring with cleft lip and/or palate (CL/P) with a frequency of 1.0–2.21 per 1000 live births [1], can cause collapse of the upper arch and influence tooth development [2,3]. Secondary alveolar bone grafting to repair the alveolar cleft was first described by Boyne and Sands [4], and focuses on stabilizing the dental arch, supporting the periodontal tissue, closing residual oronasal fistulae, and improving speech and appearance [5]. It is important for patients with CL/P to achieve success in alveolar bone grafting. However, an ‘ideal’ result is not always obtained. Retrospective studies showed that grafted cancellous bone was completely absorbed in 2–7.4% of grafted sites, and appropriate vertical bone formation was obtained only in 60–70% of cases by alveolar bone grafting [2,3,6].

The outcome of alveolar bone grafting is influenced by many factors, such as the operation timing, grafted bone sources, oral health status, and post-operative infection or inflammation [2,7]. According to previous studies, post-operative surgical wound inflammation and infection are major factors that can contribute to a greater incidence of the graft absorption, with an approximately 30% failure rate [8–10]. The opportunistic infection of surgical wounds results primarily from pathogenic bacteria or common pathogens.

Intraoral surgery, including alveolar bone grafting, should be considered a ‘clean-contaminated operative procedure’, because intraoral surgical access inevitably suffers contamination of the surgical wound with the facultative pathogenic mixed flora of the oral cavity [11]. The bacteria most frequently isolated in post-operative infections for the most part are believed to originate from the bacteria constituting the endogenous microflora of the oral cavity [12]. Additionally, gingivitis or periodontitis before the operation has been considered to contribute to the failure of alveolar bone grafting. In previous case series, approximately 20% of the failures were attributable to infection of the graft surgeries as a result of gingivitis [2]. It suggests that the oral bacterial microenvironment may be related to the post-operative inflammation of alveolar bone grafting.

In the recent studies about the cleft lip or palate repair, Rennie et al. found no significant association between immediate preoperative culture findings and the incidence of fistula formation in primary cleft palate or cleft palate fistula repair [13]. Thomas et al. also thought the preoperative microbiological culture results did not correlate with postoperative outcome [14]. These contrast with the findings of previous retrospective series, which indicated the wound infection associated with operative site colonization was an important cause of wound dehiscence and fistula formation following cleft repair [15]. Therefore, the extent to which the preoperative microbiota in fact influences operative outcomes remains a subject of debate. However, among approximately 600 predominant bacterial species in the oral cavity, about 35% are unable to be grown in culture [16]. The *16S rRNA* gene sequencing can detect the broad spectrum of both culturable and non-culturable microbiota in the oral cavity, providing insight into the diversity and community structure of the oral microbiome [17]. Given the difficulties in studying the oral microbiome using conventional, culture-based techniques, we applied *16S rRNA* gene sequencing to provide detailed insight into the oral microbiome of children with CL/P.

In recent years, next-generation sequencing is anticipated to transition from basic genomic research to clinical applications [18]. Changes in the microbiota can contribute to the pathogenesis of many diseases and reflect the health or disease state of the host [19,20]. Thus, monitoring the changes in the oral microbiota via the high-throughput sequencing is a promising potential new process for use in evaluating oral disease and prognosis [21–23].

In this study, we aimed to investigate oral microbiome profiles in children with cleft lip and palate (CLP) and to explore the pre-operative oral bacterial composition related to the prognosis of alveolar bone grafting. The saliva microbiota of subjects with inflammatory wounds were compared with those of subjects without inflammation to gain a better understanding of the oral microbial ecosystem influencing the prognosis of alveolar bone grafting.

## Materials and Methods

### Subject selection

Patients with non-syndromic cleft lip and palate (CLP) undergoing alveolar bone grafting using cancellous iliac bone were recruited from the Department of Oral and Maxillofacial Surgery at Peking University School and Hospital of Stomatology. The patients and their parents provided written informed consent, and the study followed the Declaration of Helsinki and the guidelines of the Ethics Review Committee of Peking University School and Hospital of Stomatology with regard to medical protocols and ethics (PKUSSIRB-201520028).

In total, 28 individuals, 19 boys and 9 girls ranging in age from 8 to 16 years with an average age of 10.46 years, participated in the study. Of the 28 patients, 24 had unilateral cleft lip and palate (UCLP) and four had bilateral cleft lip and palate (BCLP). All patients received the treatment of dental caries, periodontal scaling and oral hygiene instruction before the operation. They were in the healthy status of periodontal tissue without gingivitis or periodontitis. According to the clinical examination of operative sites at the third month post-operation, the individuals were divided into two groups: a non-inflammation group, which included individuals with no inflammation at the operative site (n = 15), and an inflammation group, which included individuals with inflammation (n = 13). Detailed methods were provided in supplemental methods in S1 File.

### Sample collection and microbial DNA extraction

Immediately before the operation and at the third month after the operation, 56 unstimulated saliva samples were collected from 28 individuals (two saliva samples per patient) using sterile tubes in the morning before brushing, gargling, and breakfast. Bacterial DNA from all samples was extracted using the DNeasy Blood & Tissue Kit (Qiagen, Hilden, Germany), based on the manufacturer’s protocol.

### *16S rRNA* gene amplification and MiSeq sequencing

The v3-v4 hypervariable regions of the 16S ribosomal RNA genes from the saliva samples were amplified via PCR [24]. The libraries were subjected to sequencing with an Illumina MiSeq sequencing platform using MiSeq Pair-end 300 methods [25]. The sequence data were submitted to the Short Reads Archive (Accession number SRP064362).

### Data processing and statistical analysis

The raw data generated by sequencing the 56 samples were analyzed using the pipeline tools in Quantitative Insights Into Microbial Ecology (QIIME, ver. 1.8.0), as described in the supplemental methods (S1 File) [26]. Clinical and demographic data were compared with Student’s *t*-tests. Differences in alpha diversity and the unweighted UniFrac distance between two groups, those with/without inflammation, were calculated via Mann-Whitney tests, which were also used to compare the relative abundances of individual OTUs and assigned taxa between the non-inflammation and the inflammation groups. P values < 0.05 were considered to indicate statistical significance. We calculated the Spearman’s correlation coefficient for each pair of OTUs and computed the statistical significance of the value. Edges were set between pairs of OTUs for which the Spearman’s correlation coefficient was < -0.4 or > 0.4 (P < 0.05) [27].

### Classification model based on salivary microbiota profile

We performed a supervised classification using a cross-validated random forest model, based on the pre-operative OTUs differing between non-inflammation and inflammation samples [27–29]. The relative abundances of different OTUs were processed using z-score standardization (mean 0, standard deviation 1). Three-fold cross-validation was performed on the random forest model (Weka 3.7.12, http://www.cs.waikato.ac.nz/ml/weka/index.html) using the different OTU abundance profiles with a relative abundance higher than 0.01% to assign saliva subjects to two groups (non-inflammation and inflammation).

## Results

The demographic and clinical parameters of the study subjects were shown in Table 1. No significant difference was found between the non-inflammation and inflammation groups in terms of gender, age, or cleft type. In total, 709,788 final reads were generated after filtering from the 56 saliva samples, with a mean of 12,674±6,034 (range 3,983–29,089) per sample. We ultimately detected 2,332 OTUs, with 552–1188 OTUs in individual specimens (mean 839±150), using a 99% similarity threshold based on the Greengenes database (for details, see S1 Table).

**Table 1:** Dehmographic and clinical characteristics of studied subjects.

Characteristics	Subjects (n = 28)	Grouping	
		Non- Inflammation group (n = 15)	Inflammation group (n = 13)
**- Gender**			
** Males, n (%)**	19 (67.9)	9 (60.0)	10 (76.9) ^b^
**- Age, years (range)**	10.5 ± 2.4 (8–16)	10.5 ± 2.4 (8–16)	10.5 ± 2.4 (8–16) ^b^
**- Cleft type** ^a^			
** UCLP, n (%)**	24 (85.7)	14 (93.3)	10 (76.9) ^b^
** BCLP, n (%)**	4 (14.3)	1 (6.7)	3 (23.1) ^b^
**- Cleft mucosa after operation, n (%)**			
**Edema**	13 (42.9)	0 (0)	13 (100.0)
**Serosanguineous drainage**	4 (14.3)	0 (0)	4 (30.8)
**Purulence**	0 (0)	0 (0)	0 (0)
** Wound dehiscence**	3 (10.7)	0 (0)	3 (23.1)
** Oral malodor**	8 (28.6)	0 (0)	8 (61.5)

^a^ Cleft type: UCLP = unilateral cleft lip and palate; BCLP = bilateral cleft lip and palate.

^b^ P > 0.05.

### Taxonomic composition of salivary microbiota in inflammation and non-inflammation groups

Considering the microbial taxa in the salivary samples, 9 phyla, 14 classes, 24 orders, 45 families, and 71 genera with a relative abundance higher than 0.01% were detected in the oral microbiota before and after the operation. The most abundant phyla were *Firmicutes* (an average of 38.1% of the total sequences in the inflammation group and an average of 39.3% of the total sequences in the non-inflammation group), *Proteobacteria* (31.2% in the inflammation group and 32.9% in the non-inflammation group), *Bacteroidetes* (17.8% in the inflammation group and 16.1% in the non-inflammation group), *Actinobacteria* (7.4% in the inflammation group and 7.4% in the non-inflammation group), and *Fusobacteria* (3.6% in the inflammation group and 2.5% in the non-inflammation group). These five predominant phyla constituted 98.1% of the total microbiota in the inflammation group and 98.2% in the non-inflammation group (S1 Fig). The comparison of the relative abundance of bacterial taxa between the two groups both before and after the operation were shown in the S2 Fig (for details, see the supplemental results in S1 File).

### Differences in the diversity and variation of the overall oral microbial communities between the inflammation and non-inflammation groups

The microbial diversity of the oral cavity differed significantly between the non-inflammation and inflammation groups after the operation (S3 Fig). Post-operative OTU richness, evenness, and diversity estimators were significantly higher in saliva samples from the inflammation group compared with the samples without inflammation (P < 0.05). However, the differences between the oral microbial diversity of the two groups before the operation were not statistically significant (P > 0.05, S3 Fig and S4 Table).

**Fig 1:**
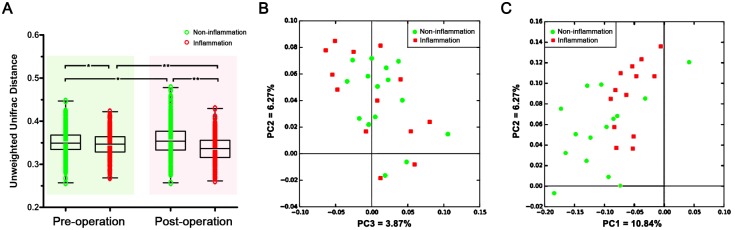
Beta diversity of the oral microbiome of non-inflammation and inflammation groups based on the unweighted UniFrac metric. (A) The inflammation samples were significantly more similar to one another than the non-inflammation samples both in the pre- and post-operative comparison. Principal coordinate analysis (PCoA) plot shows clustering of most inflammation samples, which were separated from non-inflammation samples (B) before and (C) after the operation. Red and green dots indicate inflammation and non-inflammation samples, respectively. * 0.01 < P < 0.05, ** P < 0.01.

The variation in the overall bacterial community composition, based on unweighted UniFrac distance measurements, was compared between the non-inflammation and inflammation groups. The variations in the microbial characteristics were significantly more similar in the inflammation samples than the non-inflammation samples before and after the operation (P < 0.05, Fig 1A). Principal coordinate analysis (PCoA) plots based on the unweighted UniFrac metric revealed clustering of most inflammation samples, which were separated from the non-inflammation samples before and after the operation, indicating a difference in the salivary microbial communities of the two groups (Fig 1B and 1C).

### Relative abundances of OTUs differing between inflammation and non-inflammation groups

**Fig 2:**
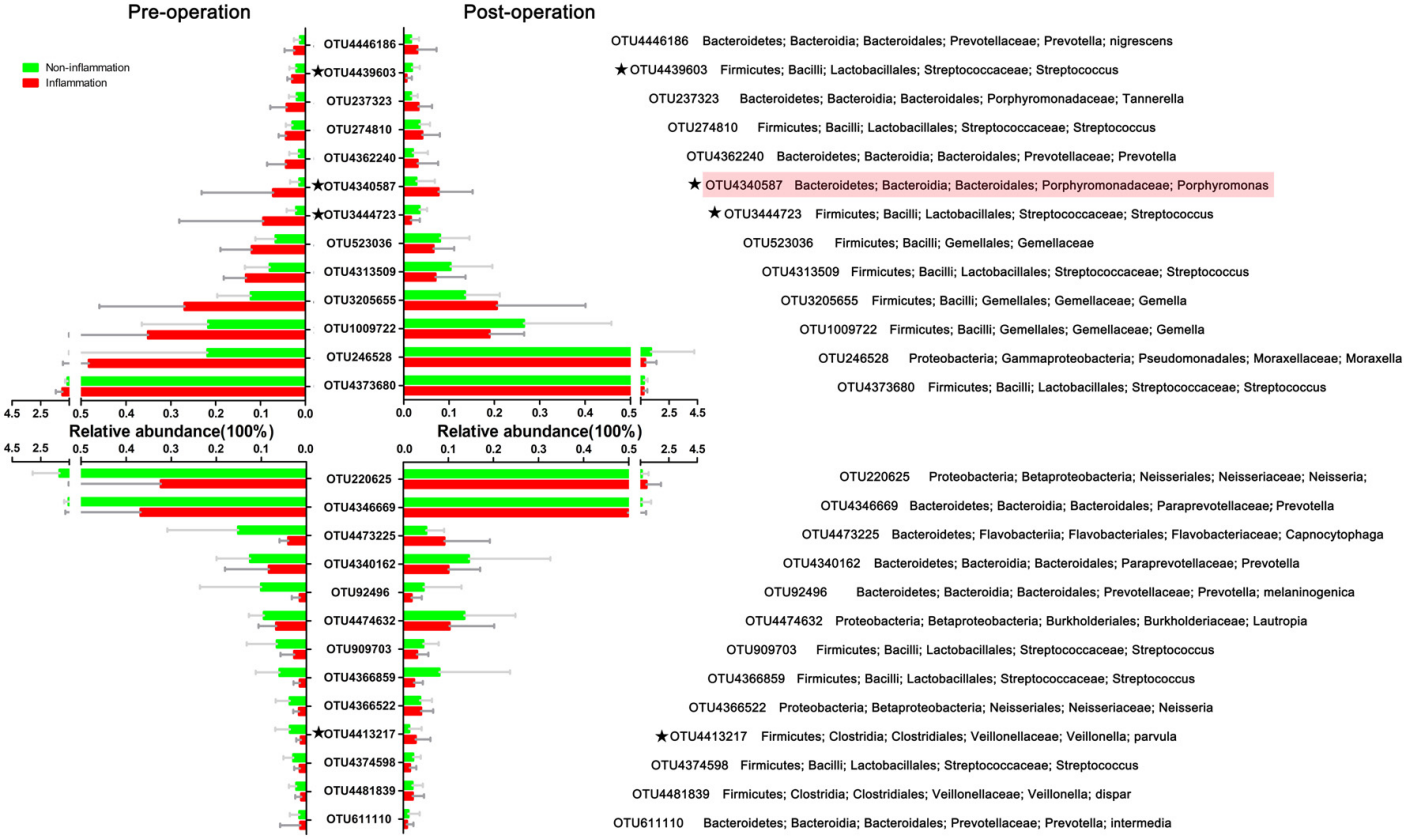
OTUs differing between the non-inflammation and inflammation groups before and after the operation. In total, 26 OTUs with relative abundances higher than 0.01% exhibited significant differences in mean relative abundances between the non-inflammation and inflammation groups before the operation (Mann-Whitney test, P < 0.05). Bars indicate means ± SEM relative abundances. The upper part of the diagram shows that the levels of 13 pre-operative OTUs were higher in the inflammation group, and the lower part shows that another 13 OTUs were lower. The relative abundance of four OTUs marked with the stars (★) were significantly different between the inflammation and non-inflammation groups both pre- and post-operation.A higher proportion of OTU4340587, corresponding to *Porphyromonas sp*. (with the pink shadow), was found in inflammation subjects than in non-inflammation subjects both pre- and post-operation.

We compared relative abundance of OTUs between the inflammation and non-inflammation groups before the operation to investigate whether inflammation status was associated with the oral bacterial composition of individuals before the operation. Analysis of the relative abundances of pre-operative bacteria showed 77 significantly different OTUs. There were 26 different OTUs with a relative abundance higher than 0.01% before the operation; 13 of these were increased in the inflammation group, and 13 were increased in the non-inflammation group (Fig 2). Among the 26 different OTUs before the operation, only four of them were differently abundant between the two groups after operation, which were marked with the star (★) in Fig 2. OTU4340587, corresponding to *Porphyromonas sp*., was observed in a higher proportion of inflammation subjects than non-inflammation subjects both before and after the operation.

### Principal component analysis showing pre-operative OTUs associated with the post-operative inflammation status

**Fig 3:**
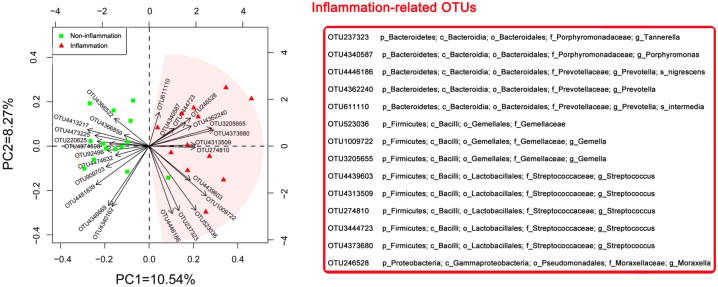
Principal component analysis (PCA) of the pre-operative OTUs information for the post-operative inflammation and non-inflammation groups. Based on 26 different OTUs of the pre-operation (relative abundance higher than 0.01%) between the two groups, the inflammation samples appeared to cluster together in the direction opposite to that of the non-inflammation samples. The 14 OTUs with the pink shadow on the right of the diagram were closely related to post-operative inflammation at the grafted sites.

Based on information about different pre-operative OTUs (relative abundance higher than 0.01%), the inflammation samples appeared to cluster together in the direction opposite to that of the non-inflammation samples (Fig 3). The inflammation-related OTUs included *Tannerella sp*., *Porphyromonas sp*., *Gemella sp*., *Moraxella sp*., *Prevotella nigrescens*, and *Prevotella intermedia*, whereas *Lautropia sp*., *Neisseria sp*., *Capnocytophaga sp*., *V*. *dispar*, *V*. *parvula*, and *Prevotella melaninogenica* were more related to the non-inflammation samples. These OTUs likely played a deciding role in dividing the groups. The OTUs corresponding to *Streptococcus sp*. and *Prevotella sp*. were present in both groups.

### Correlation between pre-operative OTUs enriched in inflammation and non-inflammation groups

**Fig 4:**
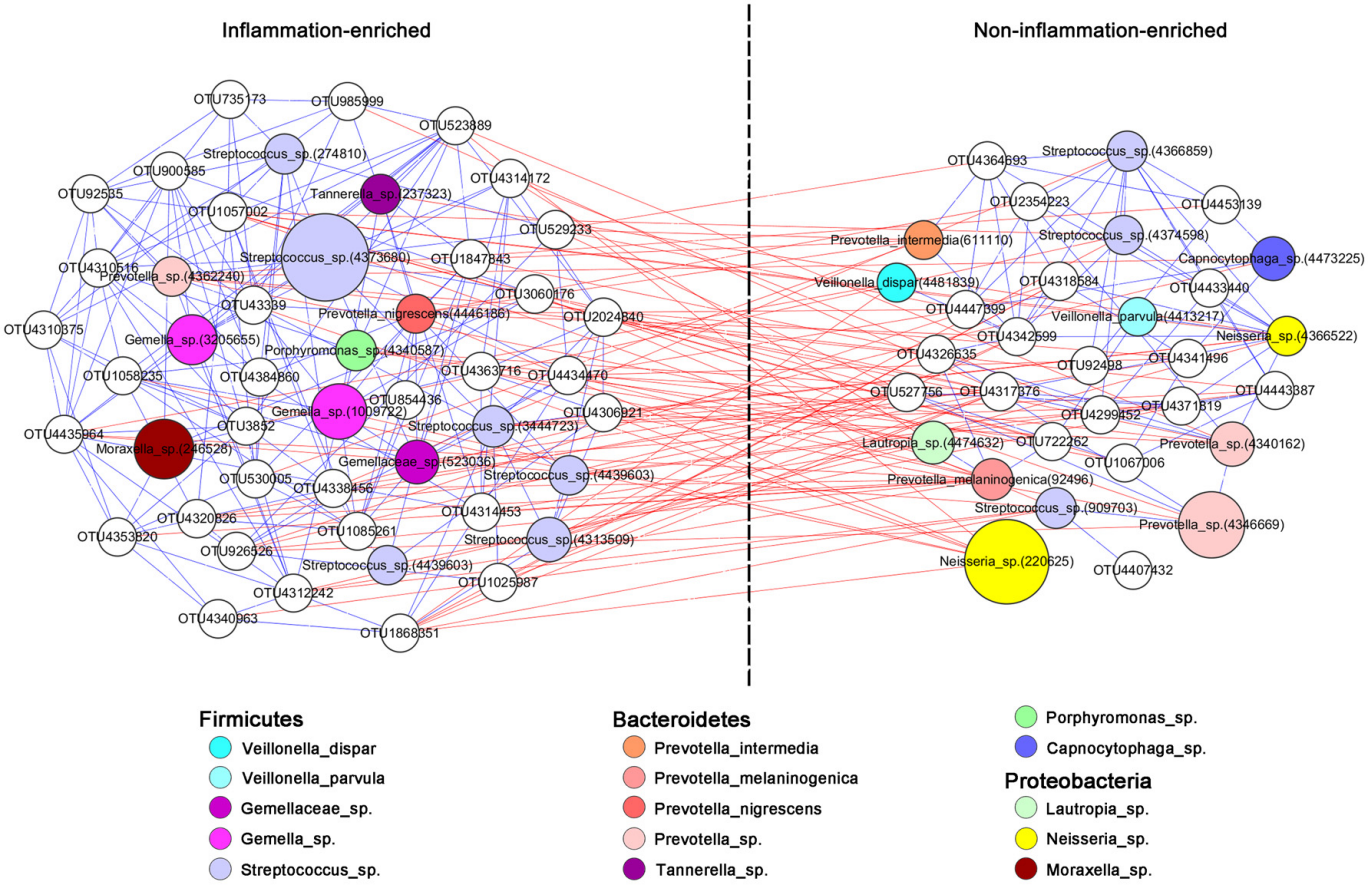
Pre-operative OTUs enriched in saliva samples of inflammation and non-inflammation subjects. Co-occurrence networks were constructed based on the relative abundance profiles of pre-operative OTUs differing between the inflammation and non-inflammation groups. Each node represents a different OTU, and the most abundant OTUs (relative abundance higher than 0.01%) are colored and annotated to indicate the species or unclassified species in a genus (sp.). The sizes of the nodes are proportional to the average relative abundances of the OTUs. Blue and red edges between each pair of OTUs indicate significantly positive and negative correlations, respectively (Spearman’s correlation coefficient > 0.4 or < -0.4, P < 0.05).

To better understand the association of the pre-operative bacteria that differed between inflammation and non-inflammation groups, we computed the correlation between the relative abundances of pre-operative OTUs in all samples. The OTUs enriched in the non-inflammation subjects showed a positive correlation (Spearman’s correlation coefficient > 0.4, P < 0.05, Fig 4), as did the inflammation-enriched OTUs, such as *Tannerella sp*., *Porphyromonas sp*., *Gemella sp*., *Moraxella sp*., and *P*. *nigrescens*. The OTUs enriched in the non-inflammation samples were negatively correlated with a number of inflammation-enriched OTUs (Spearman’s correlation coefficient < -0.4, P < 0.05, Fig 4).

### Classification of clinical status at operative sites based on microbiota profile

A cross-validated random forest model was created based on the 26 pre-operative OTUs with a relative abundance higher than 0.01% that differed significantly between the non-inflammation and inflammation groups. This classified model with three-fold cross-validation and five random trees was able to fit the post-operative status of grafted sites and yielded good classification results (S2 Table and S1 File). The cross-validated error from the random forest model was 0.3077, and the classified precision of the 28 saliva subjects was 82.14% (correctly classified instances = 23). Additionally, the sensitivity and specificity of this classified model were 76.92% and 86.67%, respectively (S3 Table). This indicated that the 26 pre-operative OTUs could, to some extent, serve as microbial indicators for prognosis of the alveolar bone grafting.

## Discussion

Secondary alveolar bone grafting is an integral part of the overall management of patients with CL/P. Post-operative surgical wound inflammation or infection is an important factor influencing the outcome of alveolar bone grafting and leads to a greater incidence of absorption of the graft [8,10]. In previous reports, about one-third of patients with failed grafts had local infection or wound dehiscence [9,10]. Post-operative wound inflammation or infection is caused by many reasons. As an intraoral surgery, alveolar bone grafting is considered a clean-contaminated surgical procedure because of the facultative pathogenic mixed flora of the oral cavity [11]. Moreover, poor oral hygiene is one of the reasons for surgical wound infections [9]. Pre-operative gingival health has been considered a major factor determining surgical success [30]. The aforementioned factors concerning post-operative inflammation/infection are also associated with the oral microbiota. This study attempted to investigate oral microbiome profiles in children with the alveolar bone grafting and identify microbial indicators for prognosis evaluation.

Based on the beta diversity of the OTU level between the non-inflammation and inflammation samples, microbial variation in the oral cavity differed significantly between the two groups both before and after the operation. The oral bacteria structure in the inflammation samples was significantly more similar than the non-inflammation samples. However, there was no significant difference between the two groups with regard to alpha diversity of oral microbiota before the operation. We suggest that the variation of bacterial OTU profiles in the pre-operative saliva may contribute to the post-operative inflammation of grafted sites.

We further compared the oral microbial composition of the non-inflammation group with inflammation group both before and after the operation. Twenty-six pre-operative OTUs with a relative abundance higher than 0.01% differed significantly between the two groups before the operation. Among these OTUs, *Veillonella parvula*, two *Streptococcus sp*., and one *Porphyromonas sp*. displayed significant difference in their post-operative relative abundance between the two groups. Only the OTU corresponding to *Porphyromonas sp*., however, was observed in a higher proportion of inflammation subjects than non-inflammation subjects both before and after the operation (Fig 2). Our data suggests that the same surgery by same surgeon has different effects on the oral microbial composition from different individuals. When the oral microbial compositions before the operation differ between the inflammation and non-inflammation samples, the alveolar bone grafting could discriminatively influence the salivary microbial community from the different groups, which respectively shift to another ones. Thus, we emphasized the analysis of the pre-operative microbiota in the oral cavity of the children with CLP.

Our analysis revealed that the inflammation-related OTUs included *Tannerella sp*., *Porphyromonas sp*., *Gemella sp*., *Moraxella sp*., *Streptococcus sp*., *P*. *nigrescens*, and *P*. *intermedia*, most of which were enriched in the inflammation group (Fig 3). These enriched microbial species are most likely residents of normal oral flora, many of which are potential opportunistic pathogens, and could contribute to the post-operation inflammation and infection.

*Prevotella* species are part of the human oral microbiota and play a role in the pathogenesis of periodontal disease and some extraoral infections, such as nasopharyngeal and odontogenic infections [31,32]. *Prevotella intermedia* is a Gram-negative, obligate anaerobic pathogenic bacterium, which is often found in acute necrotizing ulcerative gingivitis. It is commonly isolated from dentoalveolar abscesses, where obligate anaerobes predominate [33]. *Prevotella nigrescens* is part of the normal oral flora and leads to oral disease when the local tissue is infected. When *Prevotella nigrescens* colonize, they trigger an over-aggressive response from the immune system and increase the incidence of many diseases and infections [32]. *Gemella* and *Moraxella* functioning as opportunistic pathogens, are primarily found in the mucous membranes, particularly in the oral cavity, where they are capable of causing severe localized or generalized infection in previously damaged tissue [34].

In the previous studies about human microbial ecosystems, several lines of evidence have demonstrated that the role of indigenous bacteria in controlling pathogenic colonization involves preventing pathogen expansion rather than retarding exogenous acquisition [35–37]. It is also widely believed that environmental perturbations shift the balance of the oral microbiota and eventually lead to a predominance of pathogenic bacteria [29]. Thus, we suggest that a balanced oral microbiome is crucial in inhibiting the expanding of opportunistic pathogens and maintaining the stability of the microbial community, while destabilized microbial environment could potentially result in over-growth of these bacterial species and leads to increased risk of developing post-operative inflammation and infection.

Based on the co-occurring network modules in this study (Fig 4), OTUs corresponding to *Tannerella sp*., *Porphyromonas sp*., *Gemella sp*., *Moraxella sp*., *P*. *nigrescens*, and *Streptococcus sp*. were enriched in the inflammation subjects versus the non-inflammation subjects, and there were significant positive correlations between them. Furthermore, a cross-validated random forest model based on the pre-operative saliva OTUs was able to classify the post-operative status of grafted sites with high sensitivity and specificity (76.92% and 86.67%, respectively; S3 Table).

Microbiota communities in the oral cavity are polymicrobial and exist principally as biofilms on the surfaces of the teeth, gums, mucosa, and tongue [38]. Because of metabolic inter-dependencies in the oral microbial ecosystem, many oral diseases are polymicrobial infections [39,40]. Although we recognize individual bacteria as potentially pathogenic factors that modulate or damage human cells in models of infection in vivo or in vitro, a more accurate perspective is one of a pathogenic community [41]. Recent studies on the behavior of multispecies communities have shown that the presence of one or more bacteria can change virulence and gene expression in other pathogens [42–44].

*Porphyromonas gingivalis*, as one species of the genus *Porphyromonas*, is detected in low abundance in the oral cavity. However, it can cause a microbial shift in the oral cavity, allowing for uncontrolled growth of the commensal microbial community [45]. It has been proved that *Porphyromonas gingivalis* is related to increasing the virulence of other commensal bacteria both *in vivo* and *in vitro* experiments. The outer membrane vesicles of *Porphyromonas gingivalis* have also been demonstrated to be necessary for *Tannerella forsythia* to invade epithelial cells [46]. These studies indicate that the diseases associated with the human oral cavity may result from the activities of microbial communities and not only from certain individual microorganisms [41].

Although we have not determined that the measured saliva OTUs in this study could be used to predict the clinical status of grafted sites after the alveolar bone grafting because of the small sample size used in the classified model, our results do indicate that the pre-operative OTUs should be involved as a whole for the risk evaluation of the post-operative inflammation of the grafting. It is important to explore the relationship between the oral microbiota as a community and the prognosis of alveolar bone grafting. In the future, it is necessary to collect more saliva samples to confirm the accuracy of the random forest model predicting the post-operative status of grafted sites. Additionally, we will use genetic-level and metabolic-pathway analyses to explore the mechanism by which the oral microbiota profile influences the prognosis of alveolar bone grafting in further research.

## Conclusions

This study indicates that the oral bacterial community before alveolar bone grafting is related to the post-operative inflammation of grafted sites. The salivary microbial composition and variation before the alveolar bone grafting differ significantly between the postoperative inflammation and non-inflammation subjects. Among the pre-operative OTUs different between the groups, most of the inflammation-related OTUs are pathogens or opportunistic pathogens in the oral cavity. They are enriched in the oral cavities of the subjects with inflammation and show positive correlations with one another. The pre-operative oral microbial ecosystem as a whole may influence the prognosis of alveolar bone grafting.

## Supporting Information

S1 Fig Bacterial taxonomic profile of non-inflammation and inflammation groups at the phylum level, including the predominant taxa (relative abundance higher than 0.01%). (PDF)

S2 Fig The significantly different taxa with the relative abundance higher than 0.001% between non-inflammation and inflammation groups both before and after the alveolar bone grafting. (PDF)

S3 Fig Calculation of alpha diversity values for comparison of the oral microbial diversity of non-inflammation and inflammation groups before and after the operation. (PDF)

S1 File Supplemental methods and results. (DOC)

S1 Table Summaries of the background information and pyrosequencing data for all samples. (DOC)

S2 Table Detailed accuracy of the cross-validated random forest model based on the pre-operative OTUs. (DOC)

S3 Table Classified results of the cross-validated random forest model based on the pre-operative OTUs. (DOC)

S4 Table Microbial diversity estimators of the non-inflammation and inflammation groups before and after the operation. (DOC)
