# Supplementary figures and images for: Investigating Oral Microbiome Profiles in Children with Cleft Lip and Palate for Prognosis of Alveolar Bone Grafting
Source: PLoS One. 2016 May 18;11(5):e0155683. doi: 10.1371/journal.pone.0155683 (PMC4871547; doi:10.1371/journal.pone.0155683)

## Phylum

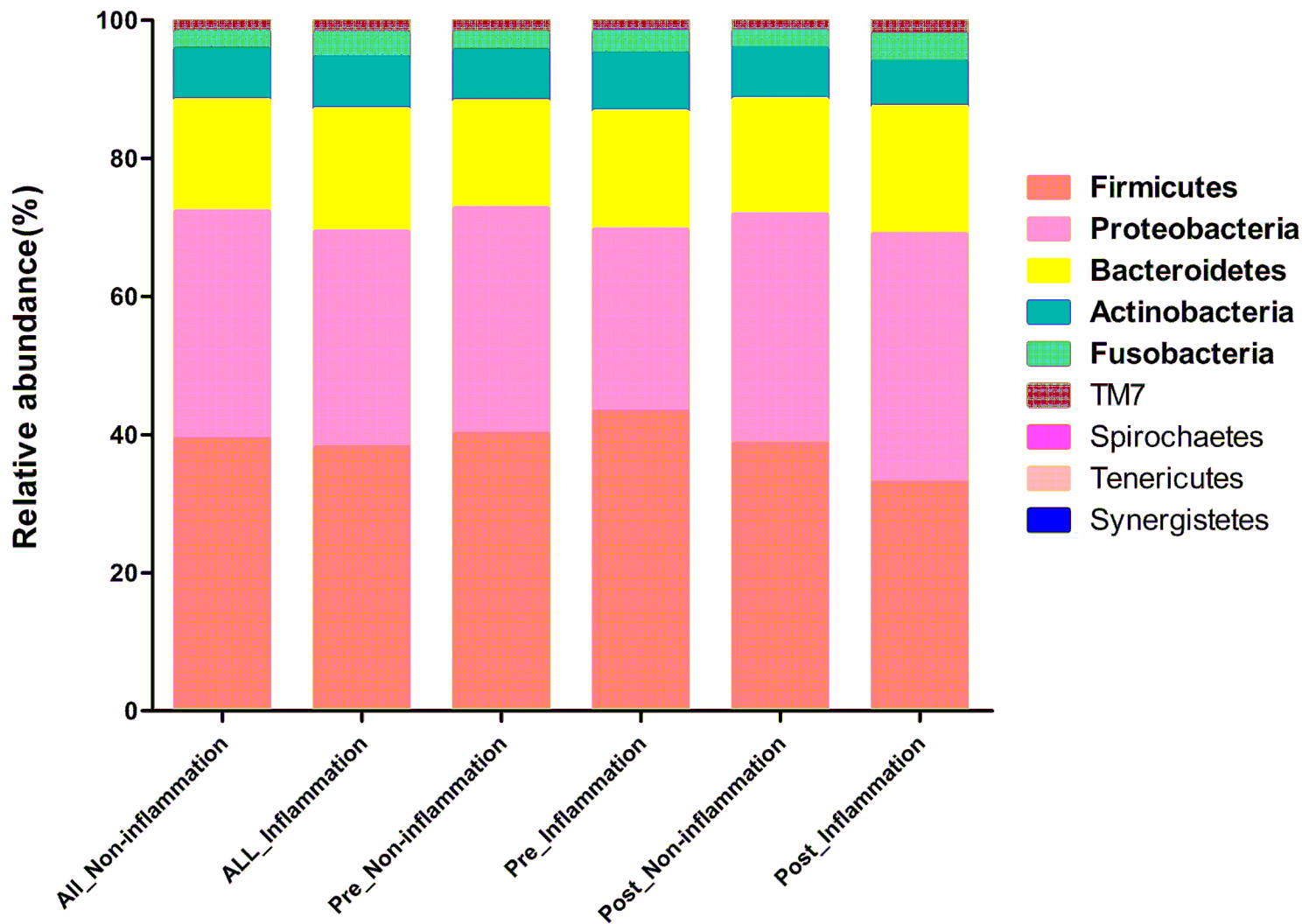

Supplement: S1 Fig — (PDF) [file pone.0155683.s001.pdf]

**Shannon**

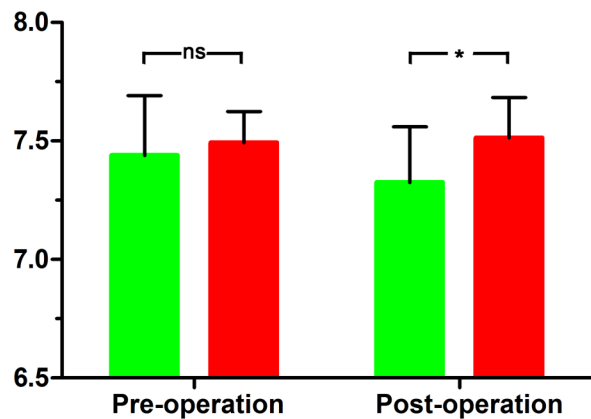

**Observed\_OTU**

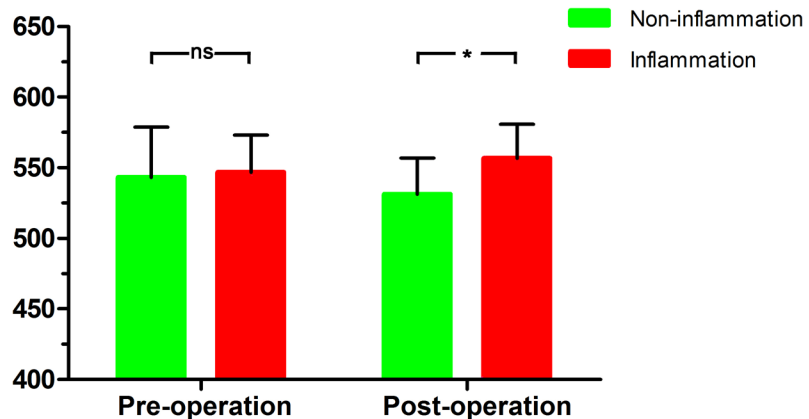

**Phylogenetic diversity**

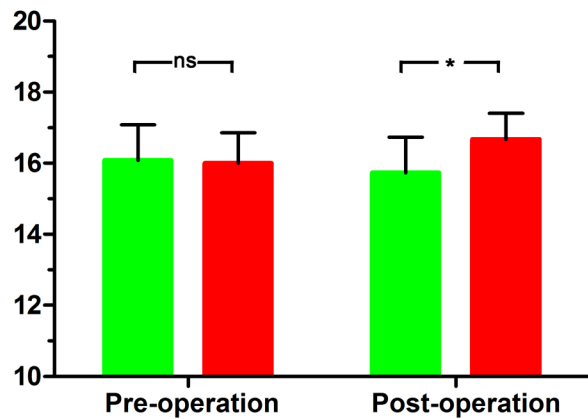

**Equitability**

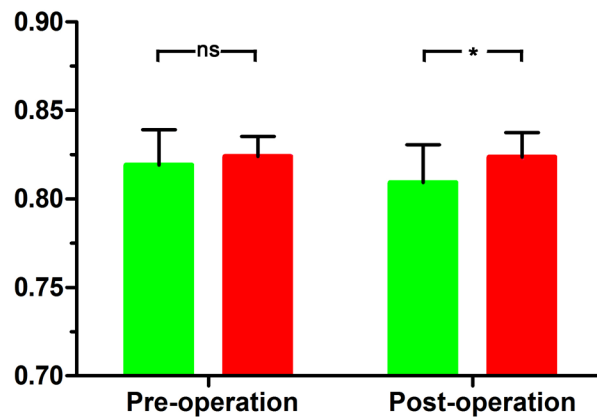

Supplement: S3 Fig — (PDF) [file pone.0155683.s003.pdf]
