# Supplementary material for: Investigating Oral Microbiome Profiles in Children with Cleft Lip and Palate for Prognosis of Alveolar Bone Grafting
Source: PLoS One. 2016 May 18;11(5):e0155683. doi: 10.1371/journal.pone.0155683 (PMC4871547; doi:10.1371/journal.pone.0155683)
Supplement: S2 Fig — (PDF) [file pone.0155683.s002.pdf]

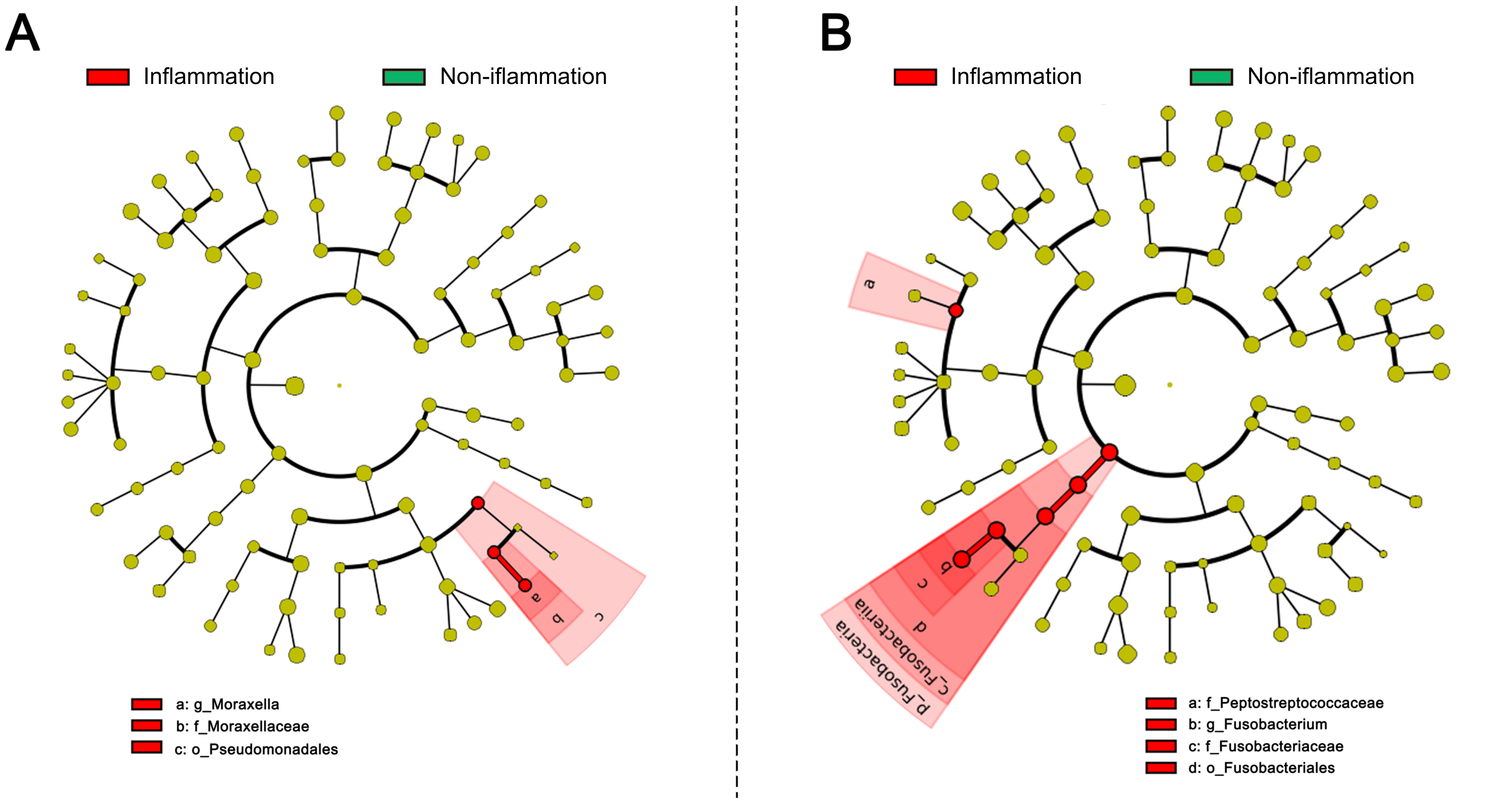

A
Inflammation
Non-iflammation
a
b
c
a: g_Moraxella
b: f_Moraxellaceae
c: o_Pseudomonadales
B
Inflammation
Non-iflammation
a
b
c
d
c_Fusobacteriia
p_Fusobacteria
a: f_Peptostreptococcaceae
b: g_Fusobacterium
c: f_Fusobacteriaceae
d: o_Fusobacteriales
