# Supplementary material for: Investigating Oral Microbiome Profiles in Children with Cleft Lip and Palate for Prognosis of Alveolar Bone Grafting
Source: PLoS One. 2016 May 18;11(5):e0155683. doi: 10.1371/journal.pone.0155683 (PMC4871547; doi:10.1371/journal.pone.0155683)
Supplement: S1 File — (DOC) [file pone.0155683.s004.doc]

**Supplemental Methods**

***Subject selection***

All patients underwent their first alveolar bone grafting, which was performed by the same surgeon using cancellous iliac bone. They were free of any medical history and long-term medication. They also had no repeated infection. No antibiotics, non-steroidal anti-inflammatory drugs, and corticosteroids were taken in every patient 1 month before the surgery. No positive signs were found in physical examination. The chest radiography was all reported normal. The results of laboratory tests were all within the normal range. All of them were medically healthy before the operation.

After the operation, all of the patients routinely received the cefoxitin sodium (intravenous injection, bid, 50mg/kg) for five days. At the third month after the operation, the operative sites of the patients were evaluated for the presence of edema, serosanguineous drainage, purulence, and wound dehiscence. When any of the above symptoms was discovered at the clinical examination, the operative site was considered inflamed. Given uniqueness of the wound purulence, which definitely arises from the specific bacteria, the subjects with the post-optative purulence were exclude. Subjects were also excluded if they had systemic diseases (except CLP) or received probiotics or antibiotics for the 1 month before sample collection.

***Sample collection and microbial DNA extraction***

At clinic visits pre- and post-operation, sterile tubes were used to collect unstimulated saliva samples from individuals in the morning before brushing, gargling, and breakfast. Next, the samples were centrifuged (12,000 rpm, 10 min) after being transported to the laboratory; they were then immediately frozen at ‑80°C until DNA extraction.

After extracting the bacterial DNA, the final concentration and quality of the DNA was measured using a Nanodrop 8000 spectrophotometer (Thermo Scientific, USA) at OD_260_/OD_280_. DNA dilutions were adjusted to ~10 ng/μL for all PCR assays.

***16S rRNA gene amplification and MiSeq sequencing***

The v3–v4 hypervariable regions of the 16S rRNA genes were amplified with the primers 5′‑GTACTCCTACGGGAGGCAGCA-3′ (forward) and 5′‑GTGGACTACHVGGGTWTCTAAT-3′ (reverse), adding sample-specific double barcodes (eight bases per barcode) before each pair of primers. Then, the PCR conditions were as follows: 3 min at 94°C, followed by 22 cycles of 94°C for 30 s, 53°C for 45 s, 72°C for 60 s, and a final 2-min extension at 72°C. The PCR products were added to a sequencing adapter according to the manufacturer’s instructions. The integrity of amplicons was confirmed with an Agilent 2100 bioanalyzer (Agilent Technology, USA), and the amplification efficiency was tested by real-time PCR (Applied Biosystems, USA). Then, the same-quality products were mixed in a group and subjected to sequencing on an Illumina MiSeq sequencing platform using MiSeq Pair-end 300 methods.

***Data processing and analysis***

The raw data generated by sequencing the salivary samples were analyzed using the pipeline tools in QIIME (ver. 1.8.0) . The forward and reverse reads in paired-end sequencing were demultiplexed using Perl scripts, based on the presence of the unique barcodes assigned to each sample. To filter low-quality sequences, those including more than 25% of nucleotides with average quality scores of < 20 were discarded. Then, the corresponding paired reads also were discarded. The 90 low-quality bases of the ends in the reverse reads were trimmed using the NGS toolkit software. The overlapping regions between the forward and the reverse paired-end sequencing reads contained at least 30 nt with at least 90% matching. Reads < 450 nt or > 480 nt were discarded. A maximum of two barcode corrections was allowed at this stage, and no ambiguous bases were permitted. After checking for chimeras and removing both the barcodes and primer sequences, the filter-passed reads were clustered into operational taxonomic units (OTUs) using a closed-reference OTU-picking strategy against the Greengenes 13_8 OTUs, at a 99% threshold . Reads were compared directly with the Greengenes database, and the reads that matched a reference sequence were clustered and assigned taxonomically against this database.

Following removal of OTUs with a single sequence and construction of the OTU table, microbial richness (Observed OTU), diversity (Shannon Index and phylogenetic diversity), and evenness (Equitability) estimators were calculated. In total, 3,900 high-quality sequences were randomly subsampled from each filter-passed dataset to estimate microbial diversity. Unweighted UniFrac distance metrics were estimated based on the OTU tables and the phylogenetic trees. Principal coordinate analysis (PCoA) was conducted according to the matrix of unweighted UniFrac distances to test the differences in saliva microbiota structure between the samples with inflammation and those without inflammation.

The differences in the relative abundance of bacterial taxa between the inflammation and non-inflammation groups both before and after the operation were performed using linear discriminant analysis (LDA) effect size (LEfSe) method (<http://huttenhower.sph.harvard.edu/lefse/>) . A significance alpha of 0.05 and an effect size threshold of 2.0 were used for logarithmic linear discriminant analysis scores.

The relative abundances of pre-operative and post-operative OTUs were compared between the non-inflammation and inflammation groups. The different OTUs before the operation were further clustered according to the Spearman’s correlation between their abundances in all samples regardless of inflammation status, and the co-occurrence network was visualized with Cytoscape (ver. 3.2.1) . To assess the classification of the oral bacteria before the operation into the non-inflammation and inflammation groups, principal component analysis (PCA) was used to show pre-operative OTUs closely related to the post-operative inflammation state.

***Classification model based on salivary microbiota profile***

For the supervised classification of the post-operative status, a cross-validated random forest model was created based on the pre-operative OTUs that differed between non-inflammation and inflammation samples . In order to enhance the prediction accuracy and interpretability of the random forest model, we used the *rfcv()* and *importance()* methods (R 3.2.1 with package Random Forest 4.6-10, <https://www.r-project.org/>) to perform the variable selection.

**References**

1. Caporaso JG, Kuczynski J, Stombaugh J, Bittinger K, Bushman FD, Costello EK et al. (2010) QIIME allows analysis of high-throughput community sequencing data. Nat Methods 7: 335-336. doi: 10.1038/nmeth.f.303 PMID: 3156573

2. Qian PY, Wang Y, Lee OO, Lau SC, Yang J, Lafi FF et al. (2011) Vertical stratification of microbial communities in the Red Sea revealed by 16S rDNA pyrosequencing. ISME J 5: 507-518. doi: 10.1038/ismej.2010.112 PMID: 3105721

3. Lif Holgerson P, Ohman C, Ronnlund A, Johansson I (2015) Maturation of Oral Microbiota in Children with or without Dental Caries. PLoS One 10: e0128534. doi: 10.1371/journal.pone.0128534 PMID: 4447273

4. Segata N, Izard J, Waldron L, Gevers D, Miropolsky L, Garrett WS et al. (2011) Metagenomic biomarker discovery and explanation. Genome Biol 12: R60. doi: 10.1186/gb-2011-12-6-r60 PMID: 3218848

5. Zhang X, Zhang D, Jia H, Feng Q, Wang D, Liang D et al. (2015) The oral and gut microbiomes are perturbed in rheumatoid arthritis and partly normalized after treatment. Nat Med 21: 895-905. doi: 10.1038/nm.3914

6. Breiman L (2001) Random forests. Machine Learning 45: 5-32. doi: 10.1023/a:1010933404324

7. Teng F, Yang F, Huang S, Bo C, Xu ZZ, Amir A et al. (2015) Prediction of Early Childhood Caries via Spatial-Temporal Variations of Oral Microbiota. Cell Host Microbe 18: 296-306. doi: 10.1016/j.chom.2015.08.005

**Supplemental Results**

***Taxonomic differences of oral microbiota between the inflammation and non-inflammation groups before and after the operation***

The comparison of the relative abundance of bacterial taxa between the two groups both before and after the operation were performed using LEfSe. The cladograms representative of the structure of the oral microbiota with a relative abundance > 0.001% and the greatest differences in taxa between the two communities were displayed in the S2 Fig. Both before and after the alveolar bone grafting, the inflammation group had a significantly higher relative abundance of bacterial taxa than the non-inflammation group (P < 0.05). The pre-operative taxa differing between the two groups mainly belonged to the *Pseudomonadales* order, *Moraxellaceae* family and *Moraxella* genus. Analysis of the post-operative taxa indicated the relative abundances of *Fusobacteria* phylum, *Fusobacteriia* class, *Fusobacteriales* order, *Fusobacteriaceae* family, *Fusodacterium* genus, and *Peptostreptococcaceae* family were higher in the inflammation group.

***Classification of clinical states at operative sites based on microbiota profile***

1. **Variable selection of random forest model using *rfcv()* and *importance()* methods**

To perform the variable selection, we used the *rfcv()* and *importance()* methods to evaluate the 26 pre-operative OTUs of which the relative abundance was significantly different between the groups.

## *1.1 The results of rfcv(step=0.95) method*

According to the table below, the results indicated that the cross-validated error was minimum when all of 26 OTUs were selected into the random forest model.

| Number of Variables | Cross-validated Error | Number of Variables | Cross-validated Error | Number of Variables | Cross-validated Error |
| --- | --- | --- | --- | --- | --- |
| **26** | **0.1071429** | 16 | 0.2500000 | 7 | 0.3571429 |
| 25 | 0.1785714 | 15 | 0.2500000 | 6 | 0.3571429 |
| 23 | 0.1428571 | 14 | 0.2857143 | 5 | 0.4285714 |
| 22 | 0.1428571 | 13 | 0.3571429 | 4 | 0.3214286 |
| 21 | 0.2142857 | 12 | 0.2857143 | 3 | 0.3928571 |
| 20 | 0.2500000 | 11 | 0.2857143 | 2 | 0.4642857 |
| 19 | 0.1785714 | 10 | 0.2857143 | 1 | 0.5357143 |
| 18 | 0.2142857 | 9 | 0.3571429 |  |  |
| 17 | 0.2500000 | 8 | 0.3571429 |  |  |

## *1.2 The results of importance() method*

The *importance()* is the definition of the variable importance measures. The results of this function indicated that the mean decrease Gini of 26 OTUs almost similar in the table below.

| Variable | Non-inflammation group | Inflammation group | Mean Decrease Accuracy | Mean Decrease Gini |
| --- | --- | --- | --- | --- |
| OTU4413217 | 2.2433752 | 2.77244636 | 3.0254702 | 0.4966315 |
| OTU4366859 | 3.6544025 | 3.65422471 | 4.7454032 | 0.5823316 |
| OTU4439603 | 1.6375689 | 3.44600703 | 3.2101412 | 0.5649197 |
| OTU4474632 | 1.7414506 | 2.35542337 | 2.5013065 | 0.4891532 |
| OTU4340162 | 3.0827041 | 3.61822815 | 4.2364009 | 0.4987816 |
| OTU4366522 | 1.4762785 | -0.06086768 | 0.9427000 | 0.4359038 |
| OTU4362240 | 3.0206343 | 3.65843688 | 3.9880763 | 0.5733705 |
| OTU4473225 | 5.4953586 | 5.01735377 | 5.9871269 | 0.5682111 |
| OTU523036 | 1.6975330 | 1.45393406 | 1.9810316 | 0.5661405 |
| OTU237323 | 0.5394774 | 1.54832937 | 1.5065951 | 0.4107937 |
| OTU220625 | 2.2396545 | 3.02651104 | 3.1644770 | 0.5586532 |
| OTU1009722 | 2.2133274 | 2.57838259 | 2.9186397 | 0.5395249 |
| OTU4313509 | -0.8828306 | 1.88027954 | 0.6546570 | 0.5350495 |
| OTU3205655 | 0.6097372 | 1.60952593 | 1.2435272 | 0.4914473 |
| OTU92496 | 5.2375528 | 6.36566211 | 6.7074310 | 0.8185885 |
| OTU909703 | 3.0614163 | 2.65221634 | 3.5251767 | 0.5635018 |
| OTU4374598 | 1.1080650 | 2.32680335 | 2.1912722 | 0.4486819 |
| OTU274810 | 0.8347511 | 0.66045120 | 0.9622590 | 0.4462206 |
| OTU246528 | 0.5560989 | 3.23004293 | 2.2800452 | 0.4337320 |
| OTU611110 | 1.8577970 | 2.70479760 | 3.2705705 | 0.3980990 |
| OTU3444723 | 1.0287062 | 4.68452803 | 3.6866994 | 0.6175961 |
| OTU4481839 | -0.1627764 | -0.52436508 | -0.2534240 | 0.4255725 |
| OTU4340587 | 2.1174710 | 2.33973640 | 3.0154217 | 0.3798014 |
| OTU4373680 | 2.4281112 | 4.44054712 | 4.5194426 | 0.5522761 |
| OTU4446186 | 0.5893639 | 0.48924046 | 0.6945246 | 0.4904030 |
| OTU4346669 | 3.3050456 | 2.51538837 | 3.4665300 | 0.4651545 |

From the results of the *rfcv()* and *importance()* methods in the variable selection, all of the 26 OTUs were very important to create the random forest model.

1. **Cross-validated random forest model based on the pre-operative OTUs differing between non-inflammation and inflammation samples**

## *2.1 Test mode: 3-fold cross-validation*

=== Classifier model (full training set) ===

Random forest of 5 trees, each constructed while considering 5 random features.

Out of bag error: 0.3077

## *2.2 All the base classifiers:*

***Random Tree 1***

==========

OTU92496 < -0.46: Inflammation (11/0)

OTU92496 >= -0.46

| OTU4366522 < -0.65: Inflammation (3/0)

| OTU4366522 >= -0.65: Non-inflammation (14/0)

Size of the tree: 5

***Random Tree 2***

==========

OTU4366859 < 0.07

| OTU4446186 < -0.52: Non-inflammation (3/0)

| OTU4446186 >= -0.52

| | OTU220625 < 1.06

| | | OTU4439603 < -1.45: Non-inflammation (1/0)

| | | OTU4439603 >= -1.45: Inflammation (14/0)

| | OTU220625 >= 1.06: Non-inflammation (1/0)

OTU4366859 >= 0.07: Non-inflammation (9/0)

Size of the tree: 9

***Random Tree 3***

==========

OTU4474632 < -0.85: Inflammation (7/0)

OTU4474632 >= -0.85

| OTU523036 < -0.09: Non-inflammation (12/0)

| OTU523036 >= -0.09

| | OTU4473225 < -0.45: Inflammation (5/0)

| | OTU4473225 >= -0.45

| | | OTU3205655 < 0.34: Non-inflammation (3/0)

| | | OTU3205655 >= 0.34: Inflammation (1/0)

Size of the tree: 9

***Random Tree 4***

==========

OTU3444723 < -0.24: Non-inflammation (14/0)

OTU3444723 >= -0.24

| OTU220625 < 0.02

| | OTU4366859 < 0.07: Inflammation (9/0)

| | OTU4366859 >= 0.07: Non-inflammation (2/0)

| OTU220625 >= 0.02: Non-inflammation (3/0)

Size of the tree: 7

***Random Tree 5***

==========

OTU4346669 < 0: Inflammation (11/0)

OTU4346669 >= 0

| OTU523036 < 0.61

| | OTU274810 < 1.8: Non-inflammation (14/0)

| | OTU274810 >= 1.8: Inflammation (1/0)

| OTU523036 >= 0.61: Inflammation (2/0)

Size of the tree: 7

## *2.3 Results of the random forest model*

=== Summary ===

Correctly Classified Instances 23 82.1429 %

Incorrectly Classified Instances 5 17.8571 %

Kappa statistic 0.6392

K&B Relative Info Score 1084.1835 %

K&B Information Score 10.8241 bits 0.3866 bits/instance

Class complexity | order 27.9645 bits 0.9987 bits/instance

Class complexity | scheme 1090.4958 bits 38.9463 bits/instance

Complexity improvement (Sf) -1062.5314 bits -37.9475 bits/instance

Mean absolute error 0.3214

Root mean squared error 0.4123

Relative absolute error 64.5051 %

Root relative squared error 82.5353 %

Coverage of cases (0.95 level) 96.4286 %

Mean rel. region size (0.95 level) 87.5%

Total Number of Instances 28

**Supplemental figure legends**

**S1 Fig. Bacterial taxonomic profile of non-inflammation and inflammation groups at the phylum level, including the predominant taxa (relative abundance > 0.01%).**

Phyla highlighted in bold were the most abundant ones in the non-inflammation and inflammation groups. Pre_Non-inflammation = the pre-operative samples of the non-inflammation group, Pre_Inflammation = the pre-operative samples of the inflammation group, Post_Non-inflammation = the post-operative samples of the non-inflammation group, Post_Inflammation = the post-operative samples of the inflammation group, All_Non-inflammation = all samples of the non-inflammation group, All_Inflammation = all samples of the inflammation group.

**S2 Fig. The significantly different taxa with the relative abundance > 0.001% between non-inflammation and inflammation groups both before and after the alveolar bone grafting.**

Taxonomic cladogram obtained from LEfSe analysis (A) before and (B) after the operation. The dots colored with the greenish represent the various levels of the taxa. The size (diameter) of each dot is proportional to the taxon’s abundance. Bacterial taxa significantly different between the inflammation and non-inflammation groups are presented in the color of the most abundant group (highlighted by small circles and by shading). Red and green dots indicate the taxa enriched in the inflammation and non-inflammation groups, respectively.

**S3 Fig. Calculation of alpha diversity values for comparison of the oral microbial diversity of non-inflammation and inflammation groups before and after the operation.**

Alpha diversity values were calculated based on a subsample of 3,900 sequences from each dataset. Differences in alpha diversity between two groups with/without inflammation were compared via Mann–Whitney test. Ns indicates P > 0.05, and * indicates P < 0.05. These estimators suggested that the richness, diversity, and evenness of oral microbial communities associated with inflammation subjects significantly increased after the operation.
