# Supplementary material for: Investigating Oral Microbiome Profiles in Children with Cleft Lip and Palate for Prognosis of Alveolar Bone Grafting
Source: PLoS One. 2016 May 18;11(5):e0155683. doi: 10.1371/journal.pone.0155683 (PMC4871547; doi:10.1371/journal.pone.0155683)
Supplement: S1 Table — (DOC) [file pone.0155683.s005.doc]

**S1** Table. Summaries of the background information and pyrosequencing data for all samples.

| Sample ID | Grafted site ^a^ | Gender | Age(yrs) | Cleft type^b^ | Pre-operation | | Post-operation | |
| --- | --- | --- | --- | --- | --- | --- | --- | --- |
|  |  |  |  |  | Reads | OTUs | Reads | OTUs |
| NI-01 | Non-inflammation | Male | 10 | UCLP | 7930 | 791 | 5588 | 573 |
| NI-02 | Non-inflammation | Male | 13 | UCLP | 19530 | 1083 | 14485 | 877 |
| NI-03 | Non-inflammation | Male | 15 | UCLP | 13118 | 875 | 9779 | 758 |
| NI-04 | Non-inflammation | Male | 9 | UCLP | 11930 | 865 | 23888 | 1039 |
| NI-05 | Non-inflammation | Female | 9 | UCLP | 9812 | 865 | 9595 | 761 |
| NI-06 | Non-inflammation | Female | 9 | UCLP | 23165 | 1010 | 9310 | 768 |
| NI-07 | Non-inflammation | Male | 16 | UCLP | 10379 | 818 | 24081 | 1124 |
| NI-08 | Non-inflammation | Female | 10 | UCLP | 6073 | 657 | 10622 | 890 |
| NI-09 | Non-inflammation | Female | 11 | UCLP | 16112 | 921 | 13452 | 868 |
| NI-10 | Non-inflammation | Male | 9 | UCLP | 9376 | 811 | 11975 | 852 |
| NI-11 | Non-inflammation | Male | 8 | UCLP | 10460 | 703 | 8673 | 785 |
| NI-12 | Non-inflammation | Male | 9 | UCLP | 15738 | 912 | 8835 | 762 |
| NI-13 | Non-inflammation | Male | 9 | UCLP | 9379 | 785 | 8342 | 700 |
| NI-14 | Non-inflammation | Female | 9 | BCLP | 19294 | 943 | 13043 | 840 |
| NI-15 | Non-inflammation | Female | 11 | UCLP | 11369 | 736 | 7400 | 617 |
| I-01 | Inflammation | Male | 8 | UCLP | 11475 | 820 | 10894 | 851 |
| I-02 | Inflammation | Female | 8 | UCLP | 28244 | 1164 | 18222 | 1003 |
| I-03 | Inflammation | Female | 13 | UCLP | 8493 | 761 | 7842 | 812 |
| I-04 | Inflammation | Male | 16 | BCLP | 4196 | 552 | 6229 | 674 |
| I-05 | Inflammation | Male | 9 | BCLP | 29089 | 1144 | 12892 | 880 |
| I-06 | Inflammation | Female | 8 | BCLP | 12729 | 893 | 8911 | 818 |
| I-07 | Inflammation | Male | 10 | UCLP | 13248 | 776 | 20621 | 1054 |
| I-08 | Inflammation | Male | 13 | UCLP | 9827 | 811 | 8654 | 733 |
| I-09 | Inflammation | Male | 9 | UCLP | 17897 | 973 | 3983 | 557 |
| I-10 | Inflammation | Male | 10 | UCLP | 26481 | 1188 | 8203 | 739 |
| I-11 | Inflammation | Male | 12 | UCLP | 14909 | 923 | 4896 | 619 |
| I-12 | Inflammation | Male | 11 | UCLP | 12940 | 880 | 16408 | 899 |
| I-13 | Inflammation | Male | 9 | UCLP | 14264 | 857 | 5508 | 602 |

^a^ Grafted site: Non-inflammation = the individuals without inflammation of the operative sites; Inflammation = the individuals with inflammation of the operative sites.

^b^ Cleft type: UCLP = unilateral cleft lip and palate; BCLP = bilateral cleft lip and palate.
