# Supplementary material for: Investigating Oral Microbiome Profiles in Children with Cleft Lip and Palate for Prognosis of Alveolar Bone Grafting
Source: PLoS One. 2016 May 18;11(5):e0155683. doi: 10.1371/journal.pone.0155683 (PMC4871547; doi:10.1371/journal.pone.0155683)
Supplement: S2 Table — (DOC) [file pone.0155683.s006.doc]

**S2 Table**. Detailed accuracy of the cross-validated random forest model based on the pre-operative OTUs

| Class | Precision | F-Measure | ROC Area | TP Rate | FP Rate | Recall | MCC | PRC Area |
| --- | --- | --- | --- | --- | --- | --- | --- | --- |
| Non-inflammation | 0.813 | 0.839 | 0.828 | 0.867 | 0.231 | 0.867 | 0.641 | 0.780 |
| Inflammation | 0.833 | 0.800 | 0.828 | 0.769 | 0.133 | 0.769 | 0.641 | 0.812 |
| Weighted Avg. | 0.822 | 0.821 | 0.828 | 0.821 | 0.186 | 0.821 | 0.641 | 0.795 |
