# Supplementary material for: Investigating Oral Microbiome Profiles in Children with Cleft Lip and Palate for Prognosis of Alveolar Bone Grafting
Source: PLoS One. 2016 May 18;11(5):e0155683. doi: 10.1371/journal.pone.0155683 (PMC4871547; doi:10.1371/journal.pone.0155683)
Supplement: S3 Table — (DOC) [file pone.0155683.s007.doc]

**S3 Table**. Classified results of the cross-validated random forest model based on the pre-operative OTUs

|  |  | Post-operative status of grafted sites | | Total |
| --- | --- | --- | --- | --- |
|  |  | Non-inflammation | Inflammation |  |
| Classified results of model | Non-inflammation | 13 | 3 | 16 |
|  | Inflammation | 2 | 10 | 12 |
| Total | | 15 | 13 | 28 |
