# Supplementary material for: Investigating Oral Microbiome Profiles in Children with Cleft Lip and Palate for Prognosis of Alveolar Bone Grafting
Source: PLoS One. 2016 May 18;11(5):e0155683. doi: 10.1371/journal.pone.0155683 (PMC4871547; doi:10.1371/journal.pone.0155683)
Supplement: S4 Table — (DOC) [file pone.0155683.s008.doc]

**S4 Table. Microbial diversity estimators of the non-inflammation and inflammation groups before and after the operation.**

| Index | | Pre-operation | | Post-operation | |
| --- | --- | --- | --- | --- | --- |
|  |  | Non-inflammation | Inflammation | Non-inflammation | Inflammation |
| Shannon | Mean | 7.439199 | 7.492354 | 7.324241 | 7.512702 |
|  | SD | 0.252485 | 0.132046 | 0.235779 | 0.170283 |
|  | *P* value | 0.492 | | 0.024^*^ | |
|  |  |  | |  | |
| Observed OTUs | Mean | 542.9667 | 546.7846 | 531.2333 | 556.6692 |
|  | SD | 35.8608 | 26.23567 | 25.73894 | 24.02122 |
|  | *P* value | 0.774 | | 0.015^*^ | |
|  |  |  | |  | |
| Phylogenetic diversity | Mean | 16.07655 | 15.9991 | 15.72635 | 16.67102 |
|  | SD | 1.001689 | 0.853686 | 1.005805 | 0.731069 |
|  | *P* value | 0.835 | | 0.013^*^ | |
|  |  |  |  |  |  |
| Equitability | Mean | 0.818998 | 0.82396 | 0.80909 | 0.82376 |
|  | SD | 0.020109 | 0.011448 | 0.021469 | 0.013818 |
|  | *P* value | 0.458 | | 0.047^*^ | |

* *P* < 0.05, Mann–Whitney test
